# Knock-Down of PRAME Increases Retinoic Acid Signaling and Cytotoxic Drug Sensitivity of Hodgkin Lymphoma Cells

**DOI:** 10.1371/journal.pone.0055897

**Published:** 2013-02-11

**Authors:** Stefanie Kewitz, Martin S. Staege

**Affiliations:** Department of Pediatrics, Martin-Luther-University Halle-Wittenberg, Halle, Germany; University of North Carolina at Chapel Hill, United States of America

## Abstract

The prognosis for patients with Hodgkin lymphoma (HL) has improved in recent decades. On the other hand, not all patients can be cured with the currently established therapy regimes and this therapy is associated with several adverse late effects. Therefore it is necessary to develop new therapy strategies. After treatment of L-540 HL cells with 5′-azacytidine (5AC), we observed increased expression of the preferentially expressed antigen in melanoma (PRAME). In addition, we detected an increased resistance of 5AC-treated cells against cytotoxic drugs. We analyzed the influence of PRAME on cell survival of HL cells by knocking down PRAME in the chemotherapy resistant cell line L-428, a cell line that express PRAME at a high level. After knock-down of PRAME using vector based RNA interference we observed increased sensitivity for cisplatin, etoposide and retinoic acid. DNA microarray analysis of HL cells after PRAME knock-down indicated regulation of several genes including down-regulation of known anti-apoptotic factors. Increased retinoic acid signaling in these cells was revealed by increased expression of the retinoic acid metabolizing cytochrome P450 (CYP26B1), a transcriptional target of retinoic acid signaling. Our data suggest that PRAME inhibits retinoic acid signaling in HL cells and that the knock-down of PRAME might be an interesting option for the development of new therapy strategies for patients with chemo-resistant HL.

## Introduction

The etiology of Hodgkin lymphoma (HL) is unknown, but immunological and molecular properties suggest that HL cells are derived from B cells [1]–[3]. HL cells have a characteristic gene-expression profile that discriminates these cells from other normal and transformed hematopoietic cells [4], [5]. With the combination of radio- and chemotherapy the majority of patients with HL can be cured. However, the established therapy is associated with a plethora of late adverse side effects and some patients with chemo-resistant disease cannot be cured [6]–[10]. Therefore it is important to search for new treatment strategies for patients with Hodgkin lymphoma.

PRAME (preferentially antigen expressed in melanoma) was identified as a tumor antigen recognized by autologous tumor-specific cytotoxic T lymphocytes from a patient with melanoma [11]. PRAME is a member of the cancer/testis antigen family and is not expressed in normal tissues except testis. This antigen is expressed in varying cancer types and PRAME expression in tumor cells has an impact on prognosis and survival of cancer patients [12], [13]. In most cases, high expression of PRAME is a marker for poor prognosis, increased development of metastasis and low disease-free survival, *e.g.* in patients with breast cancer [14]. In contrast to sensitive cell lines, chemotherapy resistant Hodgkin lymphoma cell lines show an increased expression of PRAME [5]. On the other side, high PRAME expression in childhood acute myeloid leukemia is a marker for favorable prognosis and longer survival [15]. As a result of multiple gene duplications, the human genome has multiple PRAME-homologous genes and pseudo-genes [16]. The PRAME family is present in humans and other mammals, but absent in other organisms [17]. The physiological or patho-physiological function of most members of the PRAME family is unknown. PRAME operates in the cell as a repressor of retinoic acid signaling [18]. It inhibits the retinoic acid receptor by direct binding. In normal cells in the absence of retinoic acid, repressor complexes bind to the retinoic acid receptor [13]. These co-receptor complexes have associated histone deacetylase (HDAC) activities [17]. HDAC activities change the DNA to a close conformation and inhibit transcription. When RA binds to the receptor the conformation of the ligand binding domain of the RA receptor change and a co-activator complex with associated histone acetylase (HAT) activities binds [13], [17]. The DNA conformation change to an open form and RA target genes can be transcribed, leading to differentiation, cell cycle arrest and apoptosis [17]. In cells with high PRAME expression PRAME binds to the retinoic acid receptor instead of the co-activator complex and inhibits the transcription of target genes [13]. Most of our knowledge about PRAME comes from the analysis of solid tumors or leukemia cells. All HL cell lines tested have higher expression of PRAME in comparison to normal blood cells [19]. HL cells with relatively low expression of PRAME (cell line L-540) show increased expression of PRAME after treatment with the de-methylating agent 5′-azacytidine [19]. The tumor antigen PRAME might be an interesting target for immunological treatment of HL [19], [20] but the function of PRAME in HL cells has not been elucidated. Therefore, we investigated the influence of PRAME on retinoic acid signaling and sensitivity against cytostatic drugs in HL cells.

## Materials and Methods

### Cell Lines, Cell Culture Experiments and Flow Cytometry

HL-cell lines HDLM-2, KM-H2, L-1236, L-428 and L-540 [21]–[25] were obtained from the Deutsche Sammlung von Mikroorganismen und Zellkulturen (DSMZ), Braunschweig, Germany. All cells were cultured in RPMI-1640 (Invitrogen, Karlsruhe, Germany) supplemented with 10% fetal calf serum, 100 U/mL penicillin, and 100 µg/mL streptomycin at 37°C in a humidified atmosphere with 5% CO_2_.

Cells of the line L-540 were treated in cell culture bottles at a cell density of 1×10^6^ cells/mL with 5 µM 5′-acacytidine or medium. After 3, 5, 7, 9 and 14 days, cells were harvested, RNA and DNA were isolated and the drug sensitivity of cells was determined. For this end, cells were treated for 24 hours with 60 µM roscovitine, 25 µg/mL etoposide or 25 µg/mL of cisplatin at a cell density of 500.000 cells/mL. Dead cells were identified by propidium iodide staining. The samples were analyzed on a FACScan instrument (Becton Dickinson, Heidelberg, Germany) using CellQuest Pro software (Becton Dickinson).

Expression of PRAME in HL cells was suppressed by using the BLOCK-iT POL II miR RNAi expression vector kit with EmGFP (Invitrogen, Karlsruhe, Germany) according to manufacturer’s instructions. For this end, the two oligonucleotides 5′-TGC TGA GAT GTT GTC CCT TCA TCA GCG TTT TGG CCA CTG ACT GAC GCT GAT GAG GAC AAC ATC T-3′ (top strand) and 5′-CCT GAG ATG TTG TCC TCA TCA GCG TCA GTC AGT GGC CAA AAC GCT GAT GAA GGG ACA ACA TCT C-3′ (bottom strand) were annealed and cloned into the vector pcDNA6.2-GW/EmGFP-miR. HL cells were transfected with this construct by using the CLB Kit (Biozym, Hessisch Oldendorf, Germany) and stable transfectants were selected by treatment with 3 µg/mL blasticidin.

HL cells were treated for 4 days at a cell density of 1×10^6^ cells/mL with 2.5×10^−4^ M all-*trans* retinoic acid (ATRA) or dimethyl sulfoxide (DMSO). Thereafter, RNA was isolated and the percentage of dead cell was determined by propidium iodide staining. Furthermore the drug sensitivity of cells was determined as described above. In addition, HL cells were treated at a cell density of 1×10^4^ cells/100 µL with 6.25×10^−5^ M all-*trans* retinoic acid (ATRA) or dimethyl sulfoxide (DMSO). After 4 days cell viability was assessed by 2,3-bis-(2-methoxy-4-nitro-5-sulfophenyl)-2H-tetrazolium-5-carboxanilide (XTT) assay (Roche, Basel, Switzerland) according to the manufacturer’s protocol.

### Gene Expression Analysis

RNA from cell lines was isolated using TriFast reagent (peqlab, Erlangen, Germany) following the manufacturer’s protocol. 2 µg of the RNA were transcribed into cDNA. The following primer combinations were used: actin beta (ACTB): 5′-GGC ATC GTG ATG GAC TCC G-3′, 5′-GCT GGA AGG TGG ACA GCG A-3′; B cell leukemia/lymphoma 2 (BCL2): 5′-TTC TTT GAG TTC GGT GGG GTC-3′, 5′-TGC ATA TTT GTT TGG GGC AGG-3′; CD40∶5′-ACA AAT ACT GCG ACC CCA AC-3′, 5′-CGA CTC TCT TTG CCA TCC TC-3′; early growth response 2 (EGR2): 5′-GGT CGC CTT GTG TGA TGT AG-3′, 5′-CAA ACA AAT CAG CTC CGG TA-3′; glyceraldehyde-3-phosphate dehydrogenase (GAPDH): 5′-CCA TGG AGA AGG CTG GGG-3′, 5′- CAA AGT TGT CAT GGA TGA CC-3′; interleukin 13 receptor alpha 1 (IL13RA1): 5′-AAC TTC CCG TGT GAA ACC TG-3′, 5′-AGT CGG TTT CCT CCT TGG TT-3′; preferentially expressed antigen in melanoma (PRAME): 5′- GCT GTG CTT GAT GGA CTT GA-3′, 5′- AAG GTG GGT AGC TTC CAG GT-3′; CYP26B1∶5′- GAC CCT GGA GCT GAT CTT TG-3′, 5′- TGA TGA CGC AGT CCA GGT AG-3′; tumor necrosis factor-related apoptosis inducing ligand (TRAIL): 5′-AAG GAA GGG CTT CAG TGA CC-3′, 5′-AGT TAG CCA ACT AAA AAG GCC C-3′; TRAIL receptor (TRAILR)1∶5′-AGA GAG AAG TCC CTG CAC CA-3′, 5′-GTC ACT CAA GGG CGT ACA AT-3′; TRAILR2∶5′-TGA CCT CCT TTT CTG CTT GC-3′, 5′-TAC GGC TGC AAC TGT GAC TC-3′; TRAILR3∶5′-TGT CTC CAG CCT GGC TCT AT-3′, 5′-CTC ACC CTT GTC ACC CAG TT-3′; TRAILR4∶5′-AGG CTG TTT ACA TGG GTT GC-3′, 5′-CAA ACC CTG GTC CAG TCT C-3′. Real-time quantitative RT-PCR (qRT-PCR) was performed as described using the Maxima™ SYBR Green qPCR Master Mix (Fermentas, St. Leon Roth, Germany) using the following conditions: 94°C, 30 s; 60°C, 30 s; 72°C, 45 s (40 cycles) [5], [26]. DNA-microarray analysis using Affymetrix HG_U133PLUS2.0 microarrays was performed as described [19], [26]. Data were analyzed by using expression console 1.1 (Affymetrix). MicroArray Suite 5 (MAS5) and Robust Multiarray Averaging (RMA) algorithms were applied for data analysis. Microarray data have been deposited in the Gene Expression Omnibus (GEO) data base (accession number GSE40988).

### DNA Isolation and Methylation Analysis

DNA from cell lines was isolated using the QIAamp DNA Mini Kit (Qiagen, Hilden, Germany) following the manufacturer’s protocol. 2 µg of DNA were treated with bisulfite using the EpiTect Bisulfite Kit (Qiagen, Hilden, Germany) following the manufacturer’s protocol. The following primer combinations were used [27]: PRAME non-methylated sequence: 5′-GTT GTA AGG ATG TTT TGA ATT GA-3′, 5′-CCT ACA CCA CTA CCT AAA CCA TC-3′; ALU repetitive sequence: 5′-GGT TAG GTA TAG TGG TTT ATA TTT GTA ATT TTA GTA-3′, 5′-ATT AAC TAA ACT AAT CTT AAA CTC CTA ACC TCA-3′. RT-PCR was performed using the GoTaq qPCR Master Mix (Promega, Mannheim, Germany). The reaction was performed with 10 µl GoTaq® qPCR Master Mix, 5 µl water, 1 µl of each primer combination (100 µM) and 3 µL bisulfite treated DNA using the following conditions: 94°C, 30 s; 60°C, 30 s; 72°C, 45 s (40 cycles). PCR products were subjected to agarose gel (1%) electrophoresis in the presence of ethidium bromide.

### Statistic Analysis

Statistical analysis was performed using SPSS Statistics 18 (SPSS Inc., Chicago, Ill., USA). Student’s t-test was applied for testing of significance. The same program was used for calculation of the correlation between PRAME and putative PRAME-regulated genes.

## Results

### Incubation of HL Cells with 5′-azacytidine Increases PRAME Expression

**Figure 1:**
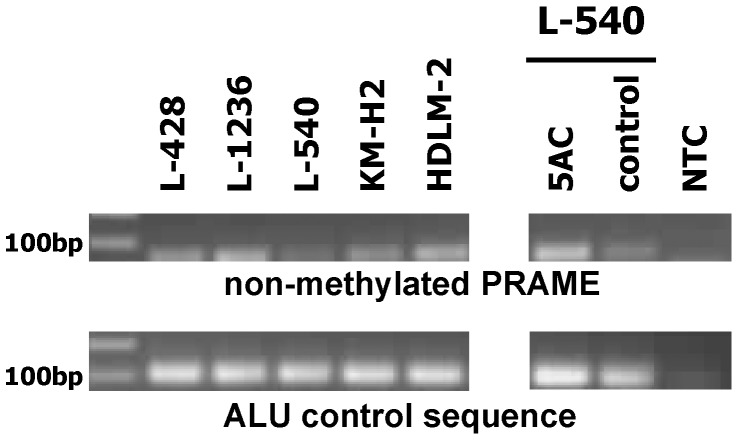
PRAME methylation status of HL cell lines. Presented is a representative methylation specific PCR with bisulfite treated DNA from HL cell lines. In the upper panel a PCR with specific primers for the non-methylated PRAME gene is presented. In the lower panel a PCR with specific primers for the ALU repetitive sequence is shown. The ALU sequence was used as control. The PCR was also performed with DNA from L-540 cells, which were treated with medium (control) and 5 µM 5AC for two weeks. Reactions without DNA template (NTC) were used as controls.

**Figure 2:**
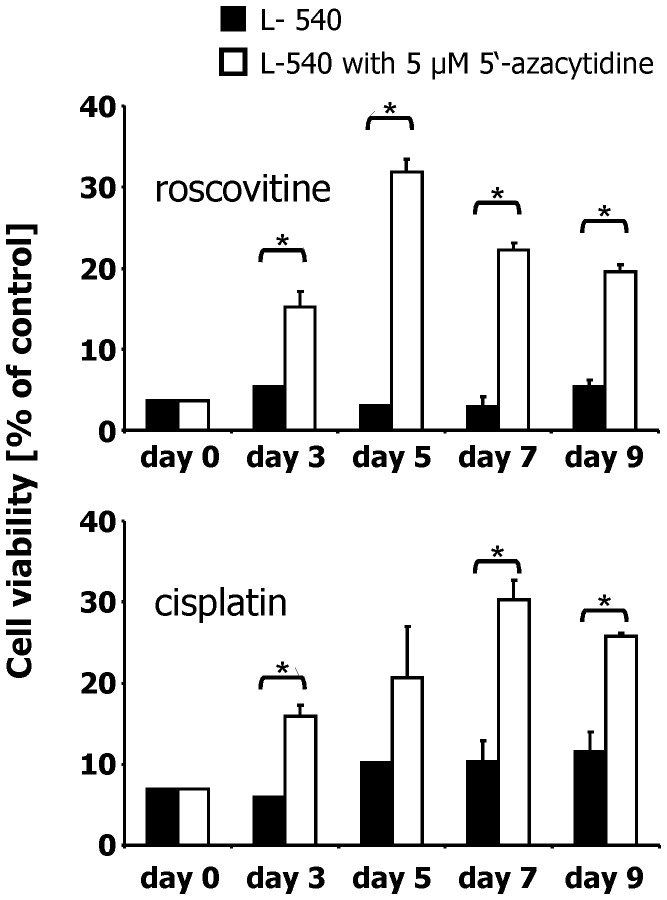
Increased sensitivity for cisplatin and roscovitin after incubation of HL cells with 5′-azacytidine. Cells of the HL cell line L-540 were incubated with 5′-azacytidine or medium. Thereafter, cells were treated with 25 µg/mL cisplatin or 60 µM roscovitin. The viability was assessed by propidium iodide staining. The number of living cells in the samples without drugs was set as 100%. Presented are means and standard errors from triplicate determinations. Asterisks indicate significance (p<0.05; Students t test).

In our previous work we observed differences in the expression of PRAME between chemo-resistant and -sensitive HL cells. We asked whether the low expression of PRAME in HL cell lines L-540 might be epigenetically regulated. Therefore, we analyzed the methylation status of PRAME in HL cell lines. We isolated DNA from HL cell lines and treated them with bisulfite. Bisulfite converts cytosine into uracil but methylated cytosines are not converted. After the conversion reaction, a methylation specific PCR was performed with specific primers for non-methylated PRAME [27]. As control we performed PCR with primers for an ALU repetitive sequence (not modified by bisulfite) [27]. A representative result is shown in Figure 1. HL cell lines L-428, L-1236, HDLM-2 and KM-H2 showed stronger signals for non-methylated PRAME than the cell line L-540. After incubation of L-540 cells with 5′-azacytidine (5AC) for two weeks we observed stronger signals for non-methylated PRAME (Figure 1). In parallel, expression of PRAME increased with time of 5AC treatment ([19] and Figure S1). We tested whether increased PRAME expression has an influence on the resistance against cytostatic drugs. We incubated the 5AC-treated cells for 24 hours with cisplatin or roscovitin, respectively. The results of these experiments are shown in Figure 2 and Figure S2. After 7 days of incubation with 5AC the expression of PRAME was 14 times higher than in the control cells; at the same time, more cells survived after treatment with cisplatin or roscovitin. We observed that the sensitivity against cytostatic drugs decreased in parallel with the increased expression of PRAME. No such decrease of sensitivity was observed when we treated constitutively PRAME expressing HDLM-2 cells with 5AC (Figure S3).

### Knock-down of PRAME Increases Sensitivity of HL Cells for Retinoic Acid and Cytotoxic Drugs

**Figure 3:**
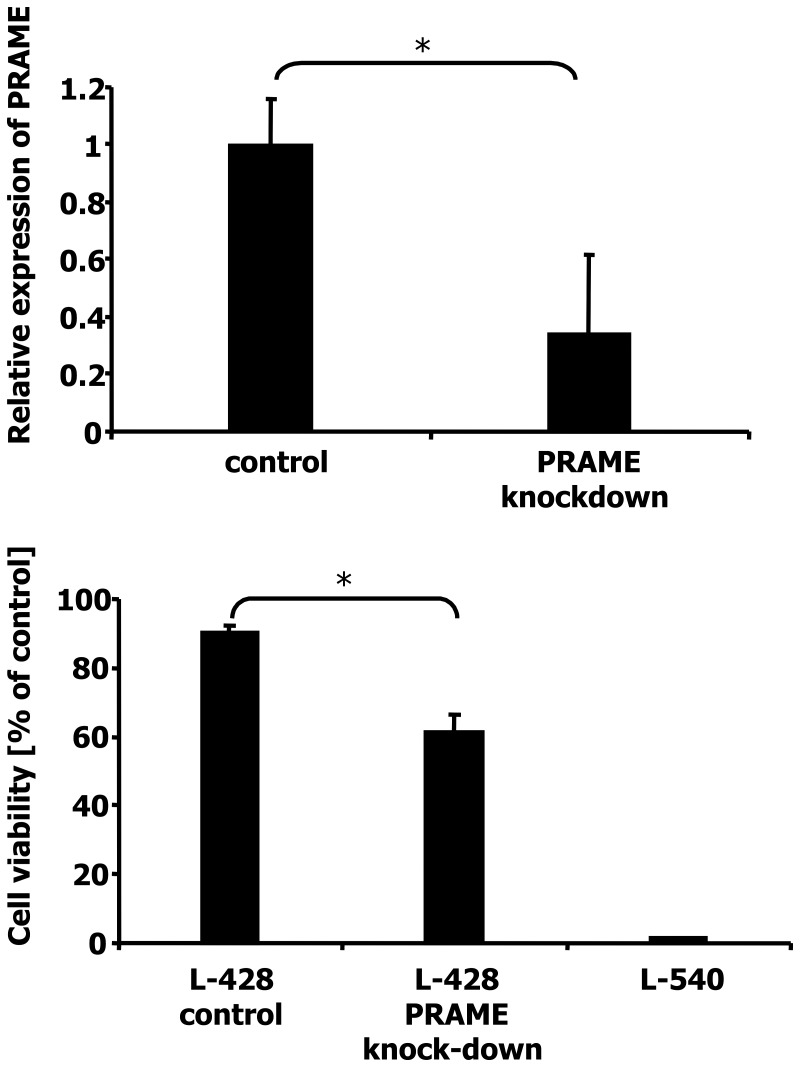
Knock-down of PRAME and increased sensitivity for retinoic acid. A) Expression of PRAME was analyzed in HL cell line L-428 by qRT-PCR. The control cells were transfected with the empty vector, whereas the other cells were transfected with a vector allowing suppression of PRAME by RNA interference. Presented are means and standard errors from six/eleven determinations. For comparative analysis, the mean of L-428 cells with the empty vector was set as 1. B) Cells of the HL cell line L-428 with empty vector, L-428 cells after knock-down of PRAME, and the HL cell line L-540 were incubated with 2.5×10^−4^ M ATRA or DMSO. After four days the viability was assessed by propidium iodide staining. The number of living cells in the samples with DMSO was set as 100%. Presented are means and standard errors from triplicate determinations. Asterisks indicate significance (p<0.05; Students t test).

It is known from other tumor models that PRAME inhibits the retinoic acid (RA) receptor [18]. In order to investigate the influence of PRAME on RA signaling in HL cells, we transfected cells of the HL cell line L-428 with a vector allowing knock-down of PRAME expression. L-428 cells express high amounts of PRAME and are resistant against RA (Figure S4). Suppression of PRAME was tested by qRT-PCR (Figure 3). Expression of PRAME in the transfected cells was 66% lower than in the control cells. The viability of the EmGFP positive cells was not decreased in PRAME knock-down cells (Figure S5). We incubated cells with low PRAME expression and control cells for 4 days with 2.5×10^−4^ M all-trans RA. Thereafter, viability was assessed by propidium iodide staining. As seen in Figure 3 and Figure S5, cells transfected with the control vector showed a viability of 90% whereas the cells with PRAME knock-down had a viability of only 61.45%. As a control we also incubated L-540 cells with ATRA. These cells express only low levels of PRAME (Figure S4) and showed the lowest viability (Figure 3).

**Figure 4:**
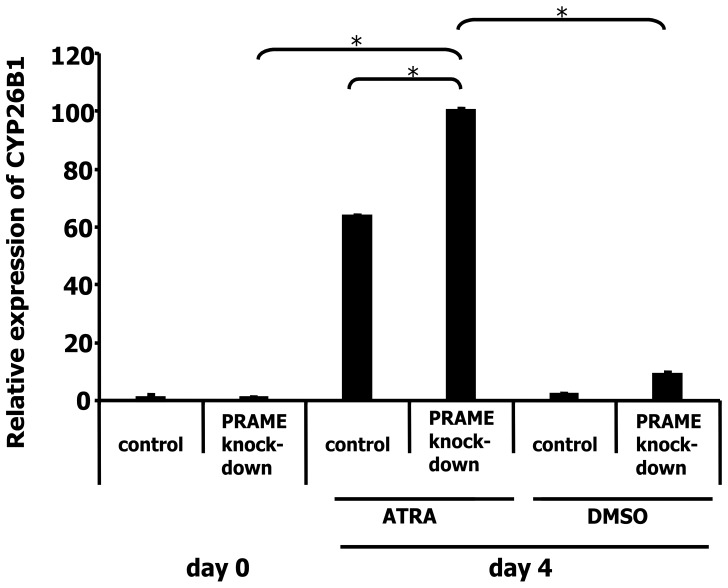
Increased induction of CYP26B1 in L-428 cells after knock-down of PRAME. Expression of CYP26B1 was analyzed in HL cell line L-428 with and without knock-down of PRAME by qRT-PCR. Cells were treated with ATRA or DMSO for four days. Presented are means and standard errors from six/eleven determinations. For comparative analysis, the means of cells on day zero was set as 1. Asterisks indicate significance (p<0.05; Students t test).

These data suggested that knock-down of PRAME increased the sensitivity for retinoic acid signaling. We analyzed the influence of PRAME on expression of RA target genes. We treated cells with knock-down of PRAME and cells with the control vector with 2.5×10^−4^ M all-trans RA. After four days we harvested the cells, isolated RNA and analyzed expression of the cytochrome P450 family member 26B1 (CYP26B1). CYP26B1 is involved in the metabolism of retinoic acid and a target of retinoic acid signaling [28]. As shown in Figure 4, expression of CYP26B1 increased after RA treatment. Control cells incubated with all-trans RA expressed 63.7 times higher levels of CYP26B1 than untreated cells. Cells with PRAME knock-down expressed 222 times higher levels, indicating that suppression of PRAME resulted in increased RA signaling.

**Figure 5:**
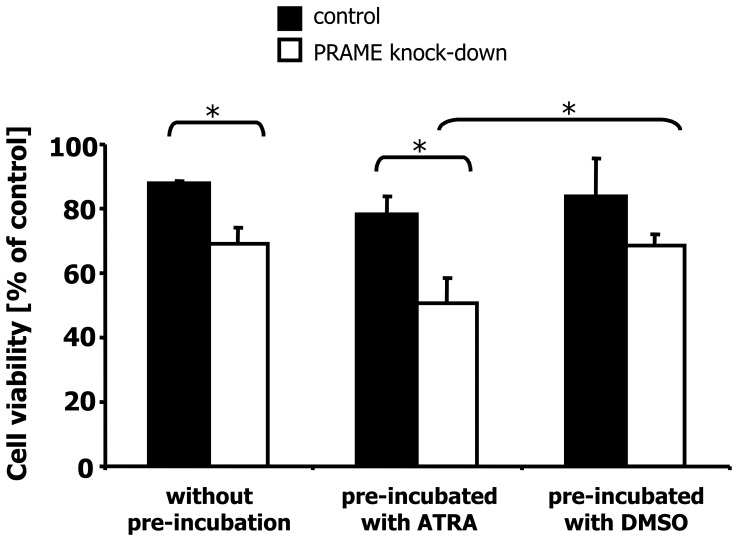
Knock-down of PRAME increased sensitivity for cisplatin and ATRA. Cells of the HL cell line L-428 after PRAME knock-down or transfection with control vector were treated for 24 hours with 25 µg/mL cisplatin. Cells were pre-incubated for four days with ATRA or DMSO and then treated for 24 hours with cisplatin. The viability was assessed by propidium iodide staining. In the experiments without pre-incubation, the number of living cells in the samples without cisplatin was set as 100%. In the experiments with pre-incubation, the number of living cells in the control cells with DMSO without cisplatin was set as 100%. Presented are means and standard errors from quadruplicate determination. Asterisks indicate significance (p<0.05; Students t test).

We asked whether PRAME knock-down influences the sensitivity for other drugs. We incubated cells with PRAME knock-down and control cells for 24 hours with 25 µg/mL cisplatin. As shown in Figure 5, the knock-down of PRAME led to an increased sensitivity for cisplatin. Cells with the control vector showed a viability of 87.8% whereas cells with PRAME knock-down show a viability of only 69%. Similar results were obtained when we treated the transfected cells with etoposide (Figure S6). Pre-incubation with RA further increases the sensitivity of cells with PRAME knock-down. Cells pre-incubated with DMSO (control) showed the same viability as cells treated with cisplatin alone. Cells pre-incubated with all-trans RA and than treated with cisplatin showed a decreased viability (Figure 5).

### Identification of PRAME Regulated Genes in HL Cells

**Figure 6:**
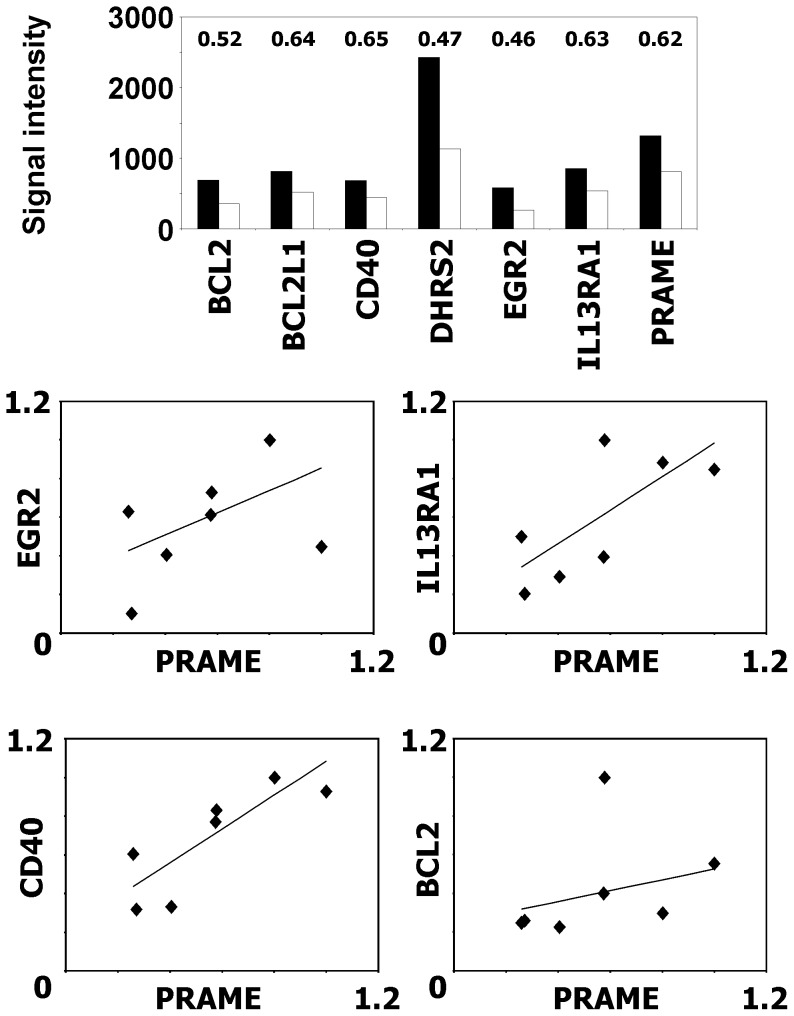
Knock-down of PRAME decreases expression of anti-apoptotic genes. Gene Expression in cells of the HL cell line L-428 after PRAME knock-down or transfection with control vector was analyzed by DNA microarray analysis. A) Presented are RMA normalized signal intensities from representative probe sets (CD40∶205153_s_at, PRAME: 204086_at, EGR2∶205249_at, BCL2∶203685_at, BCL2L1∶215037_s_at IL13RA1∶210904_s_at, DHRS2∶206463_s_at). Numbers indicate fold changes. B) Validation of differentially expressed genes by qRT-PCR. Presented are the relative expression values for four PRAME knock-down samples from three independent time points and three control samples. ACTB was used as housekeeping control and for each gene the expression in the sample with highest expression was set as one.

We asked which genes might be responsible for the increased sensitivity of L-428 cells after knock-down of PRAME. In order to identify potential candidate genes we performed a microarray analysis and compared the gene expression profile of L-428 cells that had been transfected with PRAME knock-down vector with the gene expression profile of cells that had been transfected with empty control vector. We found several genes that were differentially expressed in PRAME knock-down cells. Down regulated genes include several genes with known anti-apoptotic function, *e.g*. CD40, B cell leukemia/lymphoma 2 (BCL2), BCL2 like 1 or the interleukin 13 receptor alpha 1. From the genes shown in Figure 6, Affymetrix MAS5 detection p values of probesets for CD40 (205153_s_at and 215346_at), BCL2 (203684_s_at), and early growth response 2 (EGR2, 205249_at) increased after knock-down of PRAME. MAS5 Detection calls for CD40 (215346_at) and BCL2 changed from present to marginal (CD40) or marginal to absent (BCL2), respectively. Quantitative PCR proved down-regulation of these genes in PRAME knock-down cells (Figure 6). A significant correlation was found between expression of PRAME and CD40 (p<0.01) and IL13RA1 (p<0.05), respectively.

## Discussion

In this study we investigated the effect of PRAME on resistance of HL cells against cytostatic drugs and retinoic acid. We incubated cells with 5′-azacytidine and detected an increased expression of PRAME whereas the sensitivity of the cells for cytostatic drug decreased. Wadelin et al. also showed higher PRAME expression after incubation of cells with 5′-azacytidine [17]. In normal tissues the PRAME gene is hyper-methylated whereas the gene is hypo-methylated in malignant cells [27], [29]–[31]. Our results indicated that epigenetic changes promote high PRAME expression in chemoresistant HL cell lines. However, 5AC treatment is not specific for PRAME and additional targets might be responsible for the increased resistance of L-540 cells after treatment with this drug. Vector-based expression of PRAME in L-540 cells might be a more direct way for studying of PRAME in these cells. Unfortunately, the transfection efficiency for these cells is very low (data not shown) and until now we were not successful in establishing stable transgenic cells.

The sensitivity against cytostatic drugs decreased after treatment with 5AC. Given that in drug-resistant HL cell lines the expression of PRAME is higher than in sensitive cell lines, the results could indicated that the higher expression of PRAME is a reason for the resistance. In chronic myelogenous leukemia the over expression of PRAME is also discussed as factor for drug resistance and in diffuse large B cell lymphomas high expression of PRAME is associated with resistance against cytostatic drugs [32], [33]. When we suppressed PRAME expression using vector-based RNA interference, we observed a higher sensitivity for cytostatic drugs. These data support the assumption that PRAME plays a role in the resistance against cytostatic drugs in HL. All HL cell lines tested by PCR have higher PRAME expression than normal blood cells [19]. DNA microarray data from micro-dissected HL cells indicate expression of PRAME in a large proportion of the tumor cells. In a recently published study [34] PRAME is expressed in 24/29 samples (based on Affymetrix present calls). Interestingly, there seems to be a tendency for better outcome in patients with low expression of PRAME in this data set. However, these differences in survival rates are not statistically significant (data not shown).

PRAME is a repressor of the retinoic acid signaling pathway [18]. We investigated the effect of knock-down of PRAME on this signaling way. The HL cell line L-428 showed an increased sensitivity for all-trans RA after PRAME knock-down. The HL cell line L-540 which express only low amounts of PRAME, is very sensitive for RA. The same behavior was described for PRAME knock-down in human melanoma cells and human breast cancer cells [18]. To show that the RA signaling pathway is functional when PRAME is not expressed, we investigated the expression of CYP26B1. CYP26B1 is a target gene of retinoic acid [28]. The suppression of PRAME resulted in a strong up-regulation of this gene in HL cells. This indicates that PRAME knock-down partially restored the reactability of the cells for retinoic acid. Similarly, reduced expression of PRAME enhances the sensitivity of melanoma cells to retinoic acid [35]. Other studies show that the silencing of PRAME in primary cells increases myeloid differentiation [17].

The combination of RA and cisplatin may be an interesting option for patients with HL. We observed a decreased surviving rate of cells when we incubated them with RA followed by cisplatin. Aebi et al. observed that the combination of RA with cisplatin increased apoptosis of human ovarian adenocarcinoma cells and of squamous head and neck cancer cells [36]. Also for pancreatic adenocarcinoma cells, breast cancer cells and ovarian carcinoma cells the combination of ATRA with cisplatin has enhanced cytotoxic effect [37]–[39]. In the case of HL, such combination might be effective only for tumors with low expression of PRAME.

The function of PRAME expression in HL cells has not been clarified. Despite differences in the expression level between different cell lines, all HL cell lines express PRAME at the RNA level. In our hands, commercially available antibodies against PRAME did not detect PRAME in Western blot or immunofluorescence analysis of HL cells (data not shown). Similar results have been published by other research groups for neuroblastoma [40]. However, immunological data suggest that PRAME is expressed at the protein level in HL cells [20]. Our microarray analysis indicates that PRAME influences several genes in HL. Some of the differentially expressed genes are highly expressed in HL, *e.g.* dehydrogenase/reductase family member 2 (DHRS2). Several other genes (BCL2, BCL2L1, XIAP, CD40) have been described as apoptosis inhibiting factors. In our earlier work we observed expression of the anti-apoptotic BCL-xL isoform of BCL2L1 especially in chemo-resistant cells [5]. Recently, a link between expression of BCL-xL and janus kinase 2 (JAK2) expression in HL has been established [41]. In this study, low expression of microRNA 135a was shown to be a predictor of shorter disease free survival and relapse probability. By targeting JAK2, this micro-RNA indirectly down-regulates BCL-xL. Another interesting differentially expressed gene is CD40. The interaction between CD40 and CD40 ligand delivers an anti-apoptotic signal for HL cells by up-regulation of BCL-xL [42]. It seems unlikely that CD40 is responsible for resistance of HL cells in our *in vitro* model, because there is no CD40 ligand present. On the other side, one function of PRAME *in vivo* might be the induction of a phenotype that can respond to anti-apoptotic signals like CD40 ligand.

The pro-apoptotic or anti-apoptotic activity of DHRS2 as well as early growth response 2 (EGR2) seems to be dependent on the cellular context. For both factors anti-apoptotic effects have been described [43], [44].

In contrast to other models [45] we observed no correlation between PRAME and TRAIL in our HL model (Figure S6). Microarray data from chronic myeloid leukemia (CML) samples [46] indicate up-regulation of PRAME and concomitant down-regulation of TRAIL in CML cells in comparison to normal cells. In our analysis of HL associated gene expression profiles [5], [19], [47] we observed up-regulation of PRAME but TRAIL was not differentially expressed. The link between TRAIL and PRAME might be cell type specific. On the other hand, DNA microarray data from CML [46] suggest that, in addition to PRAME, other factors must be necessary for down-regulation of TRAIL because not all CML samples express high amount of PRAME and in-between the CML samples there is no clear correlation between PRAME and TRAIL.

In the future, the targets of retinoic acid signaling that are inhibited by PRAME in HL cells have also to be defined, *e.g.* by microarray analysis of RA treated HL cells. We used this approach successfully for the identification of RA induced genes in a neuroblastoma model [48]. With the exception of CYP26B1, we did not detect increased expression of analyzed RA target genes that we had identified in the neuroblastoma model in HL cells, indicating cell type specificity of these targets (Figure S7). The differences in the phenotype (and probably differences in the cellular origin) of different HL cell lines invites further investigations about RA induced genes in these cell lines.

Taken together, targeting of PRAME may be an interesting aspect for new therapy options for patients with HL. The down-regulation of several anti-apoptotic factors in L-428 cells after knock-down of PRAME suggests that PRAME-inhibited cells might be more sensitive for a wide range of drugs with different apoptosis inducing mechanisms. In agreement with this hypothesis, we observed altered sensitivity of our cells not only for cisplatin and etoposide but also for other small molecules like roscovitine (Figure 2). The combination of chemotherapeutics with other drugs, *e.g.* HDAC inhibitors like vorinostat [49], might further increase this effect. Indeed, we observed increased cells death of PRAME knock-down cells after incubation with cisplatin and vorinostat (Figure S8) but in our experiments these differences were not statistically significant. Our data might also have some implications for the immunological targeting of PRAME in HL [19], [20] because immune selection of antigen loss variants might concomitantly increase the sensitivity of these variants for chemotherapy [50].

## Supporting Information

Figure S1 **Increased expression of PRAME in HL cells after treatment with 5′-azacytidine.** Expression of PRAME was analyzed in HL cell line L-540 by qRT-PCR. Cells were treated with 5′-azacytidine or medium for the indicated time. Presented are means and standard error from triplicate determinations. For comparative analysis, the mean of medium-treated L-540 cells was set as 1. Asterisks indicate significance (p<0.05; Students t test).
(TIF)

Figure S2 **Increased sensitivity for cisplatin and roscovitin after incubation of HL cells with 5′-azacytidine.** Cells of the HL cell line L-540 were incubated with 5′-azacytidine or medium. Thereafter, cells were treated with 25 µg/mL cisplatin (CDDP) or 60 µM roscovitin (ROSC) or the same concentrations DMF or DMSO. The viability was assessed by propidium iodide staining. Presented are percentages of living cells from three experiments.
(TIF)

Figure S3 **5′-azacytidine treatment has no effect on roscovitin sensitivity of HDML-2 cells.** Cells of the HL cell line HDLM-2 were incubated for 5 days with 5′-azacytidine or medium. Thereafter, cells were treated with roscovitin. The viability was assessed by propidium iodide staining. The number of living cells in the samples without drugs was set as 100%. Presented are means and standard errors from three experiments.
(TIF)

Figure S4 **L-428 cells express PRAME and are resistant against retinoic acid.** A) Expression of PRAME in the indicated cell lines was assessed by quantitative RT-PCR. For comparative analysis, expression in cell line L-428 was set as one. ACTB was used as housekeeping control. B) Sensitivity of indicated cell lines for RA was assessed by XTT assay. Presented are means and standard errors from five experiments. Asterisks indicate significance (p<0.05; Students t test).
(TIF)

Figure S5 **L-428 cells express PRAME and are resistant against retinoic acid.** Cells of the HL cell line L-428 with empty vector control (left) or L-428 cells after knock-down of PRAME (right) were incubated with 2.5×10^−4^ M ATRA or DMSO. Viability was assessed in emGFP positive cells by propidium iodide staining.
(TIF)

Figure S6 **Knock-down of PRAME increases sensitivity for etoposide.** Cells of the HL cell line L-428 after PRAME knock-down or transfection with control vector were treated for 24 hours with 25 µg/mL etoposide. The viability was assessed by propidium iodide staining. The number of living cells in the samples without etoposide was set as 100%. Presented are means and standard errors from six experiments. Asterisks indicate significance (p<0.05; Students t test).
(TIF)

Figure S7 **Expression of TRAIL and other potential targets after knock-down of PRAME.** Cells of the HL cell line L-428 after PRAME knock-down or transfection with control vector were analyzed by quantitative RT-PCR (upper panel). In addition, cells were incubated with 2.5×10^−4^ M ATRA or DMSO and expression of the indicated gene were again tested by qRT-PCR (lower panel). For comparative analysis expression in control cells without RA were set as one and ACTB was used as housekeeping control. Presented are means and standard errors from three experiments.
(TIF)

Figure S8 **HDAC inhibition and PRAME knock-down in L-428 cells.** Cells of the HL cell line L-428 after PRAME knock-down or transfection with control vector were treated for 24 hours with 25 µg/mL cisplatin. Cells were pre-incubated with vorinostat or DMSO. The viability was assessed by propidium iodide staining. The number of living cells in the control cells with DMSO without cisplatin was set as 100%.
(TIF)
