# Supplementary figures and images for: Knock-Down of PRAME Increases Retinoic Acid Signaling and Cytotoxic Drug Sensitivity of Hodgkin Lymphoma Cells
Source: PLoS One. 2013 Feb 11;8(2):e55897. doi: 10.1371/journal.pone.0055897 (PMC3569423; doi:10.1371/journal.pone.0055897)

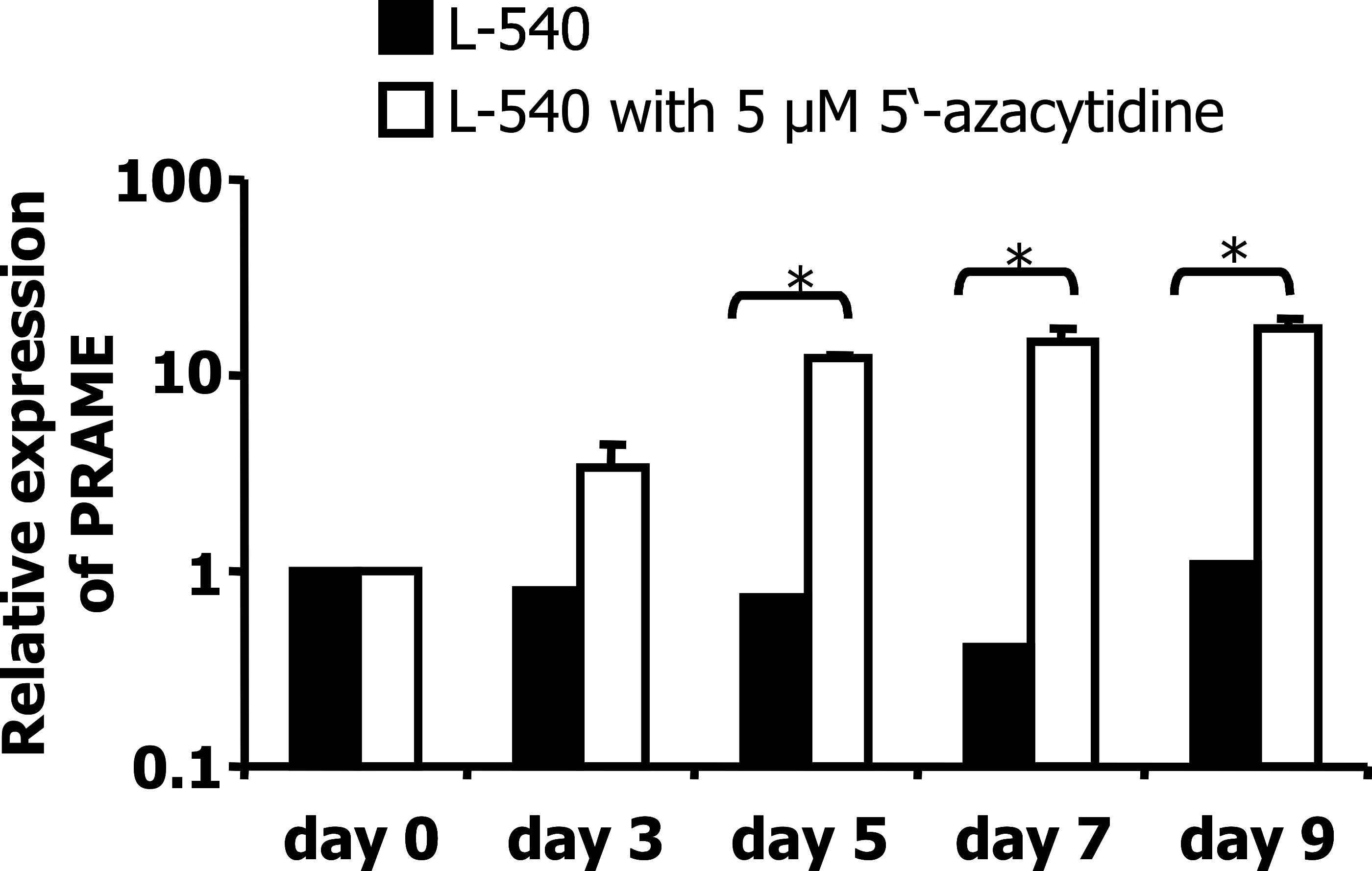

Supplement: Figure S1 — Increased expression of PRAME in HL cells after treatment with 5′-azacytidine. Expression of PRAME was analyzed in HL cell line L-540 by qRT-PCR. Cells were treated with 5′-azacytidine or medium for the indicated time. Presented are means and standard error from triplicate determinations. For comparative analysis, the mean of medium-treated L-540 cells was set as 1. Asterisks indicate significance (p<0.05; Students t test). (TIF) [file pone.0055897.s001.tif]

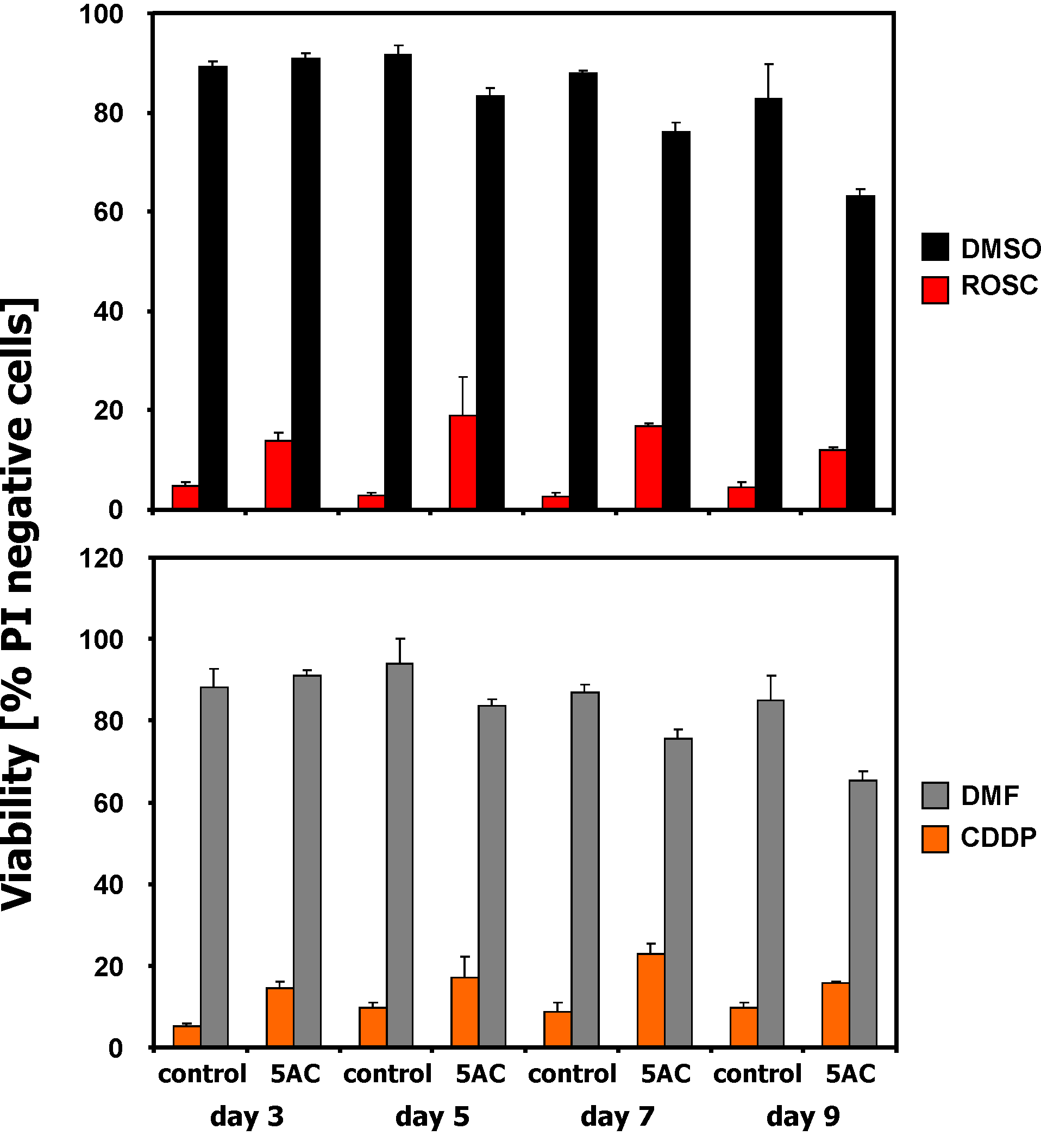

Supplement: Figure S2 — Increased sensitivity for cisplatin and roscovitin after incubation of HL cells with 5′-azacytidine. Cells of the HL cell line L-540 were incubated with 5′-azacytidine or medium. Thereafter, cells were treated with 25 µg/mL cisplatin (CDDP) or 60 µM roscovitin (ROSC) or the same concentrations DMF or DMSO. The viability was assessed by propidium iodide staining. Presented are percentages of living cells from three experiments. (TIF) [file pone.0055897.s002.tif]

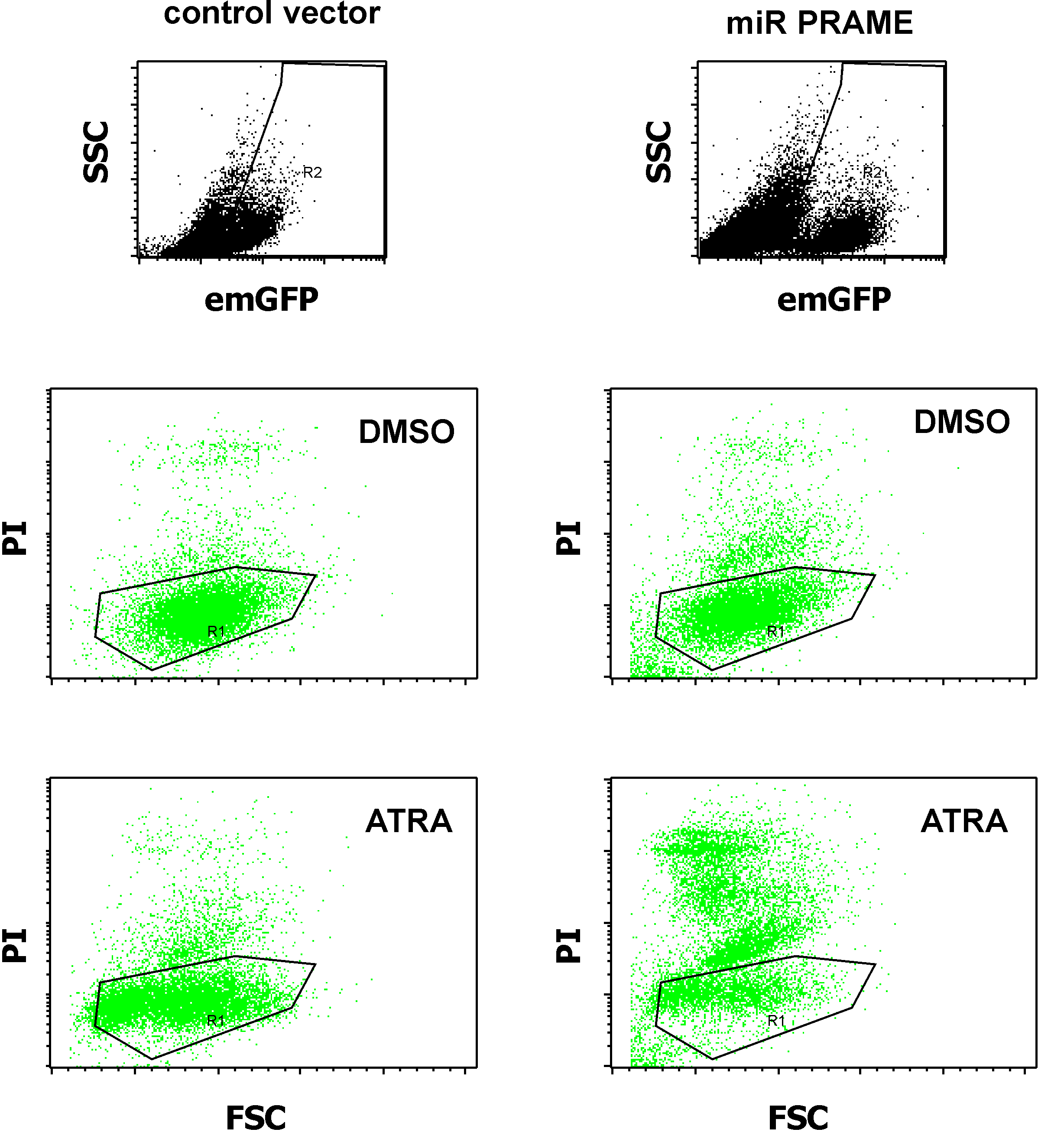

Supplement: Figure S5 — L-428 cells express PRAME and are resistant against retinoic acid. Cells of the HL cell line L-428 with empty vector control (left) or L-428 cells after knock-down of PRAME (right) were incubated with 2.5×10^−4^ M ATRA or DMSO. Viability was assessed in emGFP positive cells by propidium iodide staining. (TIF) [file pone.0055897.s005.tif]

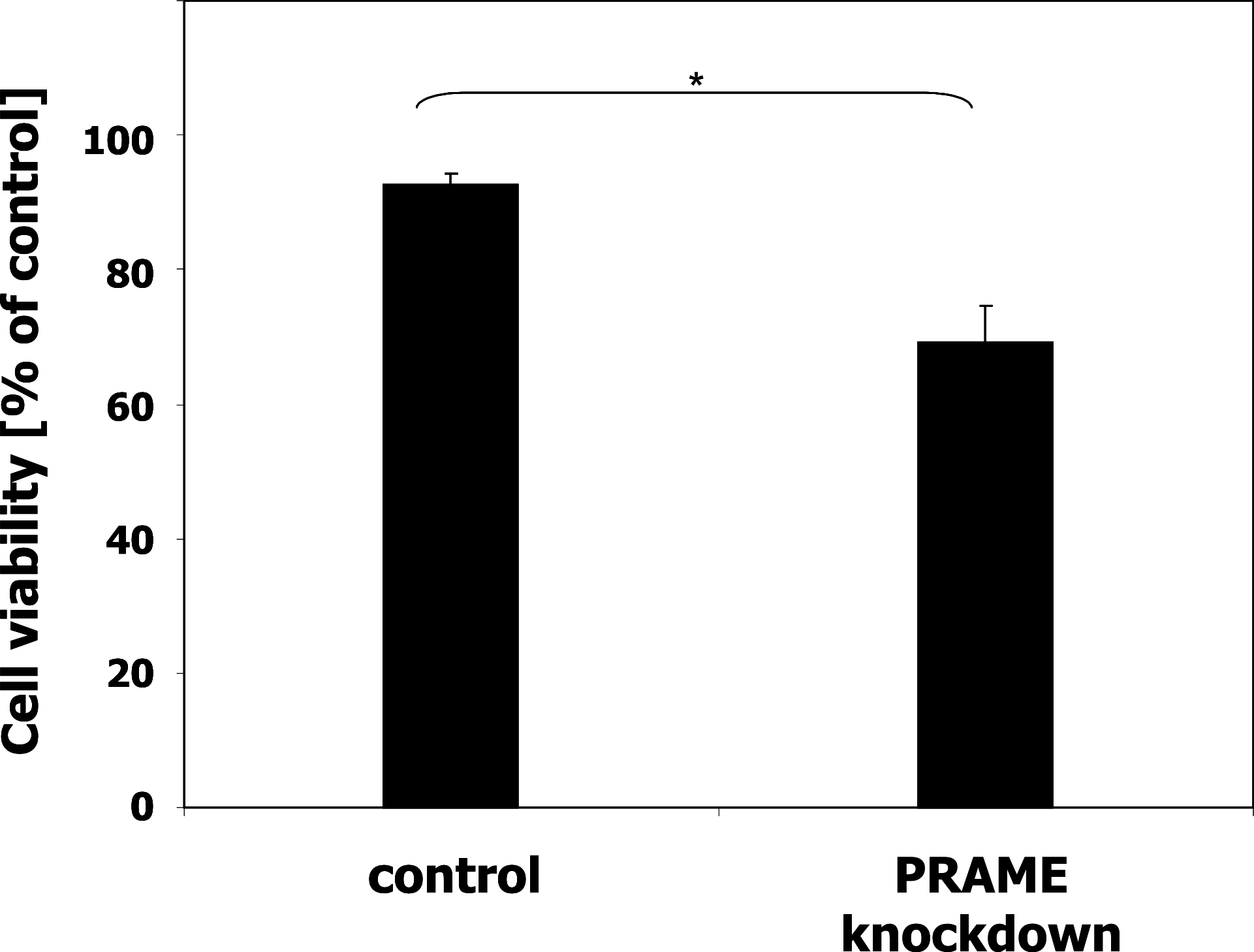

Supplement: Figure S6 — Knock-down of PRAME increases sensitivity for etoposide. Cells of the HL cell line L-428 after PRAME knock-down or transfection with control vector were treated for 24 hours with 25 µg/mL etoposide. The viability was assessed by propidium iodide staining. The number of living cells in the samples without etoposide was set as 100%. Presented are means and standard errors from six experiments. Asterisks indicate significance (p<0.05; Students t test). (TIF) [file pone.0055897.s006.tif]

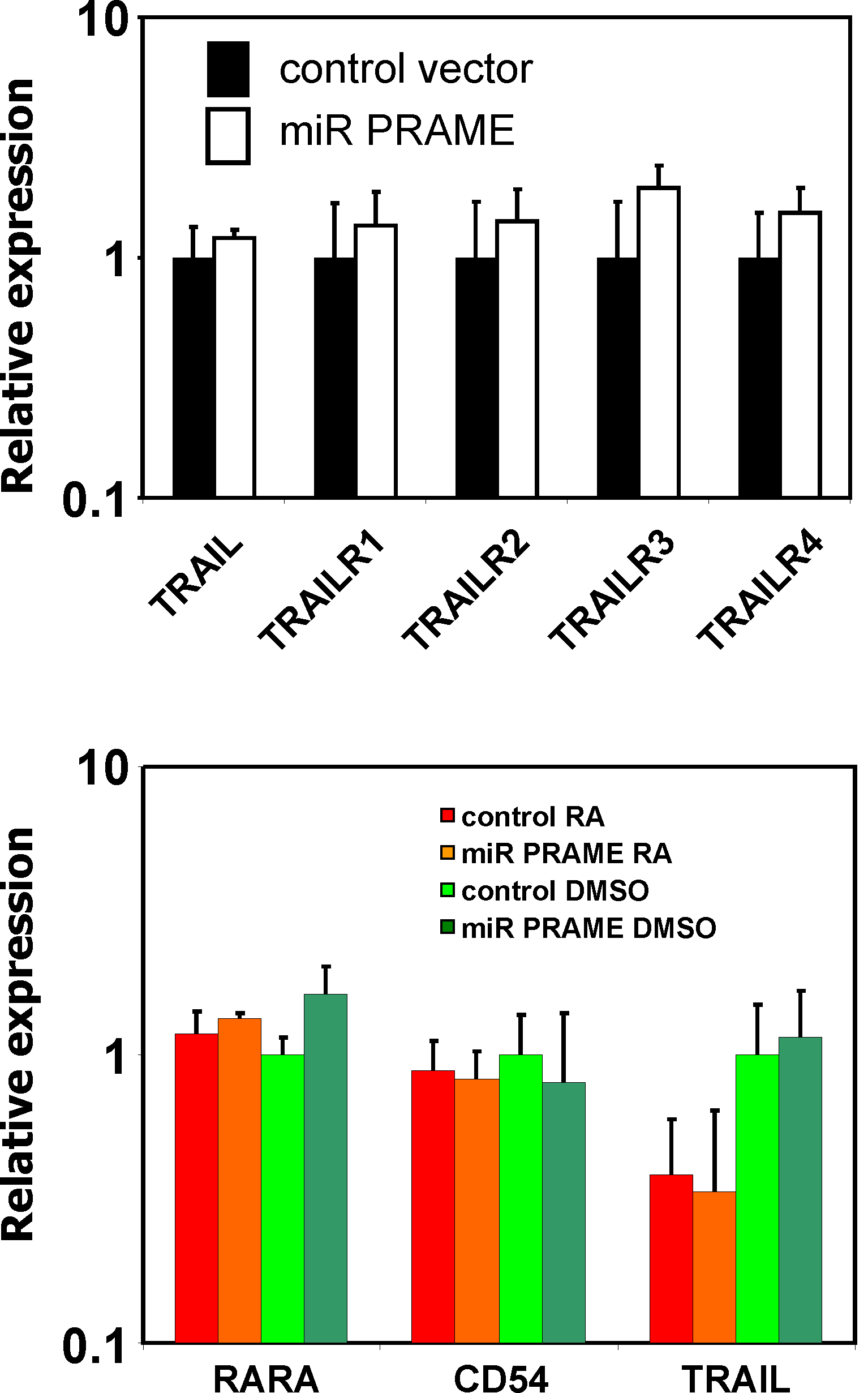

Supplement: Figure S7 — Expression of TRAIL and other potential targets after knock-down of PRAME. Cells of the HL cell line L-428 after PRAME knock-down or transfection with control vector were analyzed by quantitative RT-PCR (upper panel). In addition, cells were incubated with 2.5×10^−4^ M ATRA or DMSO and expression of the indicated gene were again tested by qRT-PCR (lower panel). For comparative analysis expression in control cells without RA were set as one and ACTB was used as housekeeping control. Presented are means and standard errors from three experiments. (TIF) [file pone.0055897.s007.tif]

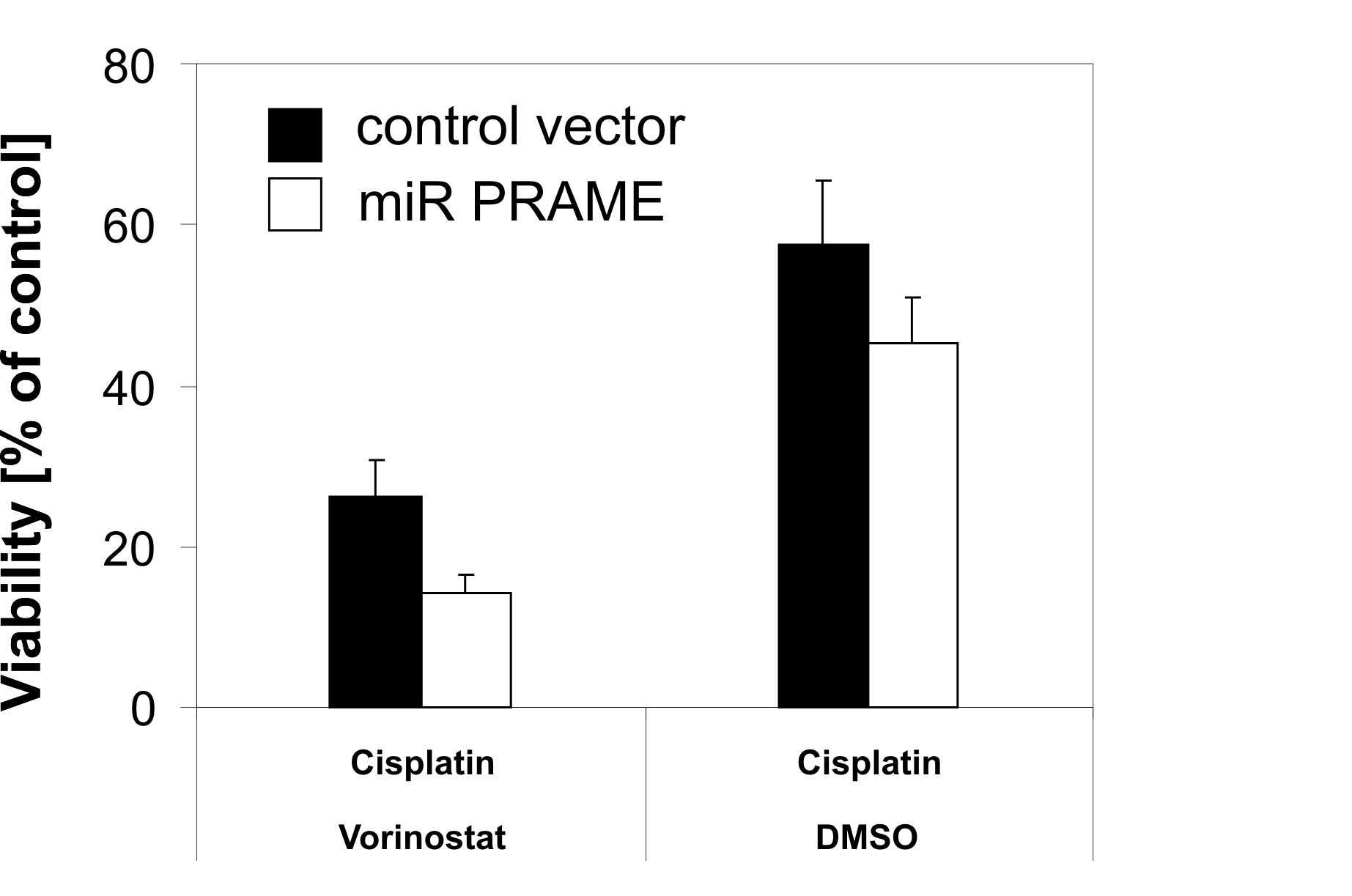

Supplement: Figure S8 — HDAC inhibition and PRAME knock-down in L-428 cells. Cells of the HL cell line L-428 after PRAME knock-down or transfection with control vector were treated for 24 hours with 25 µg/mL cisplatin. Cells were pre-incubated with vorinostat or DMSO. The viability was assessed by propidium iodide staining. The number of living cells in the control cells with DMSO without cisplatin was set as 100%. (TIF) [file pone.0055897.s008.tif]
